# Epidemiology, Immunology and Clinical Characteristics of COVID-19 (EPIC^3^)—Database of a prospective longitudinal observational study within the Veterans Health Administration

**DOI:** 10.3389/fpubh.2025.1535315

**Published:** 2025-06-02

**Authors:** Liuye Huang, Xumin Li, Samin I. Kamal, Jonathan D. Sugimoto, Cindy H. Liu, Tracy Wang, Daniel K. Morelli, Jordanna B. Midthun, Vivek R. Pakanati, Katrina V. Deardorff, Jennifer L. Sporleder, Jude Lopez, Mark Holodniy, Nicholas L. Smith, Jennifer S. Lee, Javeed A. Shah, Jennifer M. Ross

**Affiliations:** ^1^Veterans Affairs Puget Sound Health Care System, Seattle, WA, United States; ^2^Department of Epidemiology, School of Public Health, University of Washington, Seattle, WA, United States; ^3^International Vaccine Institute, Seoul, Republic of Korea; ^4^Veterans Health Administration, Public Health National Program Office, Washington, DC, United States; ^5^Veterans Affairs Palo Alto Health Care System, Palo Alto, CA, United States; ^6^School of Medicine, Stanford University, Stanford, CA, United States; ^7^Division of Allergy and Infectious Diseases, Department of Medicine, University of Washington, Seattle, WA, United States; ^8^Department of Global Health, University of Washington, Seattle, WA, United States

**Keywords:** SARS-CoV-2, Veterans, epidemiology, longitudinal cohort, database

## Introduction

The health burden caused by SARS-CoV-2 infection has been devastating nationally and internationally (1, 2). Over the past 4 years, much research has been conducted to characterize the clinical trajectory of acute SARS-CoV-2 infection and to identify risk/prognostic factors for infection and disease severity, respectively (3–6). Despite the end of the COVID-19 public health emergency declaration in the U.S. (7) and globally (8) in May 2023, research into COVID-19 continues. Priorities have shifted from understanding acute manifestations to long-term sequelae, clinical phenotypes, persistence of vaccine immunity, and identification/validation of clinically relevant biomarkers, among others (9–20). Such shifts underscore the need for prospective and longitudinal data, collected using externally validated and widely adopted instruments across participants with heterogenous sociodemographic and clinical characteristics to allow for research across various domains of interest.

Therefore, the Cooperative Studies Program (CSP) of the Veterans Health Administration (VHA), U.S. Department of Veterans Affairs (VA) authorized the Epidemiology, Immunology, and Clinical Characteristics of COVID-19 (EPIC^3^) study—a prospective, multi-site, cohort study conducted within the nation's largest integrated healthcare system. The design of the EPIC^3^ study is based on that of the Department of Defense (DoD)-sponsored Epidemiology, Immunology, and Clinical Characteristics of Emerging Infectious Diseases with Pandemic Potential (EPICC-EID) study (21). The EPIC^3^ study collects information regarding patient reported outcome measures (PROMs) and biospecimens across pre-defined visit time windows, over a follow-up period of 24 months. These data are augmented with real-world data (RWD) sourced from participants' VA electronic health records (EHRs) to address the following aims:

(1) To identify sociodemographic, clinical, virologic, and immunologic factors which influence the trajectory of SARS-CoV-2 infection, with a particular emphasis on understanding medium and long-term sequelae, and prognostic factors associated with adverse outcomes.
(2) To characterize factors associated with and protective against initial infection, reinfection, and breakthrough infection after vaccination.
(3) To identify both individual and facility-level risk factors for SARS-CoV-2 infection, among Veterans living in community living centers (CLCs)—long-term skilled nursing facilities managed by the VA.

## Methods

### Registration

This study is registered on ClinicalTrials.gov (NCT05764083). More details regarding the registration can be found at https://clinicaltrials.gov/study/NCT05764083#study-overview.

### Study design and setting

The EPIC^3^ study is a VHA-based, prospective, longitudinal observational study of U.S. Veterans with and without SARS-CoV-2 infection. It consists of 3 prospectively followed sub-cohorts: inpatient, outpatient, and CLC. The study recruited participants from June 10th, 2020 to September 30th, 2022 at 16 VA medical centers across the U.S.: Baltimore, MD; Boston, MA; Cleveland, OH; Dallas, TX; Denver, CO; Durham, NC; Gainesville, FL; Little Rock, AR; Milwaukee, WI; Palo Alto, CA; Philadelphia, PA; Portland, OR; Salt Lake City, UT; San Antonio, TX; Seattle, WA; and West Haven, CT. There are 2 years of active follow-up that include up to 10 questionnaire and biospecimen data collection time points. Each participating VA medical center operates as a local study site, recruiting participants into at least one of the three sub-cohorts. The Seattle Epidemiologic Research and Information Center (ERIC) monitors and coordinates enrollment and study operations across the sites. The Public Health Research Center (PHRC) located at the VA medical center in Palo Alto, CA processes, manages, and archives study specimens. The Seattle ERIC is the steward of the data repository, biospecimen repository, and the registry of participants who have consented to being contacted about future studies.

### Study population

The study enrolled U.S. military Veterans with and without SARS-CoV-2 infection who utilized services at VHA facilities. For the inpatient and outpatient sub-cohorts, infection status was confirmed by the record of a positive reverse transcriptase polymerase chain reaction (RT-PCR), antibody, or antigen test for SARS-CoV-2 in the VHA EHR. Self-reported RT-PCR tests conducted outside the VA were permitted as well, but this contingency was not often used. In the inpatient and outpatient sub-cohort, 430 participants (58.4%) and 1,280 participants (66.9%) had confirmed SARS-CoV-2 positive status, respectively. For each positive participant enrolled in the inpatient and outpatient sub-cohorts, the study attempted to enroll a Veteran who sought the same type of care (inpatient versus outpatient) at the same site but without the record of a positive SARS-CoV-2 test within the 21-days preceding the date of seeking care. All residents of participating CLCs were eligible to enroll regardless of their SARS-CoV-2 infection status at enrollment.

### Recruitment and consenting

#### Recruitment

Recruitment procedures differed among the inpatient, outpatient, and CLC sub-cohorts. There was no predetermined sample size for any sub-cohort; the recruitment goal was to consent as many participants as possible given the site staffing capabilities and the prevalence of SARS CoV-2 infection.

##### Inpatient sub-cohort

Every 24 h, the research team used VHA EHRs to find newly eligible inpatient participants. Research staff were also able to identify eligible participants. Clinical staff approached inpatients with confirmed or suspected SARS-CoV-2 infection to discuss the study's goals and to determine if the patient was capable of providing informed consent. Clinical staff referred interested inpatients to the site's research team, who then scheduled a recruitment and consent discussion.

##### Outpatient sub-cohort

At VHA SARS-CoV-2 testing locations, study flyers were posted or distributed by VHA staff. Every 24 h, the research team used the nightly-updated VHA EHR to find newly eligible outpatient participants who sought care or had a test ordered at participating locations. The research team then reached out to potential outpatient participants by phone and mailed a recruitment package that included an invitation letter and a study description. Interested outpatients were also invited to contact the research team directly using the information on the recruitment flyers posted at public locations at VHA facilities.

Lists of eligible participants were then loaded into a study database that all study sites used for recruitment and enrollment. The date on which an eligible participant was identified from the VHA EHR was defined as their index date. In both the inpatient and outpatient sub-cohorts, study staff were allotted 72 h from index date to make first contact with eligible SARS-CoV-2 positive participants, with eligible participants then being allowed an indefinite amount of time to make their decision regarding participation.

For each sub-cohort except for CLC, the study sought to recruit a 1:1 ratio of SARS-CoV-2 positive and negative participants. To achieve the 1:1 ratio, study sites were instructed to consult the study database for eligible SARS-CoV-2 negative patients for each enrolled SARS-CoV-2 positive counterpart, matching on study site and sub-cohort. Study sites were also instructed to prioritize enrolling SARS-CoV-2 negative matches who had the closest duration of elapsed time from the time of seeking care to the time of recruitment to their positive counterparts. For enrolled SARS-CoV-2 positive participants, the time since seeking care was defined as the number of hours that have elapsed between the start of their eligibility period and their recruitment into the study. While for SARS-CoV-2 negative participants, it indicated the number of hours that have elapsed between the start of their eligibility period and when the study database was consulted to find a matched SARS-CoV-2 negative participant.

##### CLC sub-cohort

For residents at CLC sites, the local research team, in collaboration with CLC staff, informed all residents and their family members about the study through letters. Interested Veterans or their legally authorized representative were approached for informed consent. Definition for SARS-CoV-2 status in CLC was same for other two cohorts, although it was not part of the recruitment criteria.

#### Consenting

Informed consent was required and obtained prior to recruitment for each participant. Paper and secure digital documentation of written informed consent were permitted. As part of the informed consent process, the site principal investigators or their designee provided a plain-language description of the study's aims, procedures, likely risks, and benefits, as well as describing rights of the participant and which study components are optional. Participation in the study's data and biospecimen repositories were required; participation in the participant registry was optional. In cases where a Veteran had impaired capacity to make informed decisions, the outlined process remained the same, incorporating a legal authorized representative (LAR) or personal representative (PR) to provide consent and a PR to provide HIPAA authorization.

#### Ethical and safety considerations

This study was approved by the VHA Central IRB (VHA Central IRB Study Number 20–14). Given the observational nature of the study, participants were determined to be at minimal risk for any study-related adverse outcomes. All the electronic data, written information, and materials are stored securely behind the VHA security firewall.

### Data collection and management plan

#### Data collection

After enrollment, baseline data were collected via questionnaires and biological sampling. Inpatient and outpatient participants were asked to complete follow-up questionnaires and provide biologic samples for testing during follow-up visits on days 3, 7, 14, 21, and 28, as well as months 3, 6, 12, 18, and 24 after enrollment. CLC participants were asked to complete questionnaires and provide biospecimens every 3 months until 24 months after enrollment. CLC participants who were discharged from a CLC were offered the option of continuing to participate in the study by continuing with the inpatient/outpatient data collection schedule, starting with the next scheduled visit relative to their enrollment date. EHR data were abstracted for each participant in both a retrospective and prospective manner (Table 1).

**Table 1:** Overview of questionnaires, clinical data, and biological sampling at each time point.

**Questionnaires and biospecimens collected for each sub-cohort**	**Visit time** ^**d**^													
	**D0**	**D3**	**D7**	**D14**	**D21**	**D28**	**M3**	**M6**	**M9**	**M12**	**M15**	**M18**	**M21**	**M24**
**Inpatient sub-cohort**														
Baseline questionnaire (modified MVP)	✓	-	-	-	-	-	-	-	-	-	-	-	-	-
Symptoms questionnaire (Flu-related)	✓	✓	✓	✓	✓	✓	✓	✓	-	✓	-	✓	-	✓
Vaccination questionnaire	✓	✓	✓	✓	✓	✓	✓	✓	-	✓	-	✓	-	✓
Long-Term Symptoms questionnaire	-	✓	✓	✓	✓	✓	✓	✓	-	✓	-	✓	-	✓
Nasopharyngeal and oropharyngeal swabs^a^	✓	✓	✓	✓	✓	✓	-	-	-	-	-	-	-	-
Blood^b^ and saliva^c^	✓	✓	✓	✓	✓	✓	✓	✓	-	✓	-	✓	-	✓
**Outpatient sub-cohort**														
Baseline questionnaire (modified MVP)	✓	-	-	-	-	-	-	-	-	-	-	-	-	-
Symptoms questionnaire (Flu-related)	✓	✓	✓	✓	✓	✓	✓	✓	-	✓	-	✓	-	✓
Vaccination questionnaire	✓	✓	✓	✓	✓	✓	✓	✓	-	✓	-	✓	-	✓
Long-Term Symptoms questionnaire	-	✓	✓	✓	✓	✓	✓	✓	-	✓	-	✓	-	✓
Nasopharyngeal and oropharyngeal swabs^a^	✓	✓	✓	✓	✓	✓	-	-	-	-	-	-	-	-
Blood^b^ and saliva^c^	✓	✓	✓	✓	✓	✓	✓	✓	-	✓	-	✓	-	✓
**CLC sub-cohort**														
Baseline questionnaire (modified MVP)	✓	-	-	-	-	-	-	-	-	-	-	-	-	-
Symptoms questionnaire (Flu-related)	✓	-	-	-	-	-	✓	✓	✓	✓	✓	✓	✓	✓
Vaccination questionnaire	✓	-	-	-	-	-	✓	✓	✓	✓	✓	✓	✓	✓
Long-Term Symptoms questionnaire	-	-	-	-	-	-	✓	✓	✓	✓	✓	✓	✓	✓
Blood^b^	✓	-	-	-	-	-	✓	✓	✓	✓	✓	✓	✓	✓

^a^Swab collections include one or more biospecimens of the following types: Nasal swab, Nasopharyngeal swab, Oropharyngeal swab.

^b^Blood collections include one or more biospecimens of the following types: Peripheral blood mononuclear cells (PBMC), Ethylenediaminetetraacetic acid (EDTA) Plasma, Sodium Citrate Plasma, Sodium Citrate Buffy Coat, PAXgene Blood RNA Tube, Serum (venous), Serum (capillary).

^c^Saliva collections include one or more biospecimens of the following types: Saliva (with preservative), Saliva (without preservative).

^d^ D means “Day”; M means “Month”.

##### Questionnaires

A modified Million Veteran Program (MVP) COVID-19 survey was administered at baseline (22). This survey assessed SARS-CoV-2 and COVID-19 risk factors related to sociodemographic characteristics, substance use and lifestyle behaviors, family history, sources of exposures to infection, and medical and mental health comorbidities. The survey also assessed the impact of COVID-19 on health-related quality of life.

A flu-related symptoms questionnaire, a vaccination questionnaire, and a long-term symptoms questionnaire were administered at baseline and/or at multiple follow-up time points. The flu-related symptoms questionnaire addressed the preceding 24-h period at each visit. The licensed FLU-PRO instrument (23, 24) [license obtained through an agreement between Leidos Biomedical Research, Inc. and the VHA Cooperative Studies Program (CSP)] formed the primary component of this survey, with additional questions assessing current mental acuity and the impact of clinical interventions on infection. The vaccinations questionnaire (25) collected patient-reported history of receiving SARS-CoV-2 vaccination, along with requesting permission to contact the administering non-VHA medical provider, if the participant could not remember sufficient details about vaccine doses received. Lastly, the long-term symptoms questionnaire was administered during all follow-up visits after the initial enrollment visit. It consisted of a set of validated questions for the assessment of health-related quality of life (EQ-5D-5L) (26) and sequelae of SARS-CoV-2 infection, including symptoms of fatigue (PROMIS fatigue 6a) (27, 28), shortness of breath (modified MRC Dyspnea Scale) (29), and cognitive function (PROMIS cognitive function 4a) (30, 31).

Participant responses were recorded using Research Electronic Data Capture (REDCap), a secure, HIPAA compliant, web-based application for creating and managing online surveys and databases. REDCap at VHA is hosted on the VHA Informatics and Computing Infrastructure (VINCI) and maintained by the VHA Information Resource Center (VIReC), with enhanced security features. The survey questions are available on the CSP #2028 website: https://www.seattle.eric.research.va.gov/research/CSP-2028-EPIC3/Data-sources.asp.

##### Clinical data

A wide array of information can be captured from EHRs at the VHA Corporate Data Warehouse (CDW), including medical history of conditions relevant to SARS-CoV-2 and COVID-19 risk, SARS-CoV-2 vaccinations received, vital signs, diagnosis codes for medical comorbidities, hospitalization records, and other data relevant to clinical care and treatment. Clinical indices calculated using EHR data include the Charlson Comorbidity Index (CCI) (32) and the Veterans Affairs Severity Index for COVID-19 (VASIC) (33). The VASIC is a 4-category (mild, moderate, severe, or death) measure of the maximum illness severity within 30 days of enrollment that was calculated for SARS-CoV-2-positive inpatients.

##### Biospecimens

Biospecimens were collected at in-person visits and, if in-person was not feasible, through participant self-collection. At in-person visits, the study collected peripheral blood in whole blood ethylenediaminetetraacetic acid (EDTA) tubes or sodium citrate tubes, serum separator tubes, RNA-stabilizing PAXGene^®^ tubes, and CPT™ mononuclear cell preparation tubes. Saliva and the eluant from nasal or nasopharyngeal swabs was collected at in-person visits for inpatient and outpatient participants. Residual clinical samples from inpatients were also collected as available. Specimens were then shipped to the biorepository and laboratory at PHRC within 24-h at 3–4 degrees Celsius. Most study sites were also able to have an additional set of blood specimens assayed by on-site Pathology and Laboratory Medicine Service (PLMS) laboratories.

Where an in-person visit was not feasible, devices for self-collecting saliva (Spectrum Solutions SDNA-1,000) and TASSO-SST^®^ or Tasso^®^ plus kits for collecting a small amount of capillary blood were shipped to the participant, which they were asked to self-administer and mail free-of-charge back to PHRC. Study staff were available to provide real-time, telephone-based coaching to help interested participants through the self-collection process. Specimens collected by these self-administered kits were stable at ambient temperature for at least a week.

#### Data management plan

Quality control activities were performed throughout data collection, including duplicate entries, outliers, and consistency checks within REDCap, and periodic operational checks to minimize data entry errors and deviation of protocol requirements. Multiple validated assays were used by PHRC to analyze study-collected specimens including SARS-CoV-2 RT-PCR, enzyme-linked immunosorbent assays (ELISA) for SARS-CoV-2 nucleocapsid and spike IgG antibodies, whole genome sequencing of SARS-CoV-2 viruses detected by RT-PCR, and multiplex cytokine/chemokine assays. Assay results from biospecimens will be stored in a PHRC biorepository and inventory management system. In addition, assays requested of PLMS laboratories include: complete blood cell count (CBC) with differential, including platelets, hematocrit, hemoglobin, white blood count with differential; complete metabolic panel, including albumin, calcium, sodium, potassium, chloride, carbon dioxide, blood urea nitrogen (BUN), creatinine, glucose, total and direct bilirubin, aspartate aminotransferase (AST), alanine aminotransferase (ALT), alkaline phosphatase, total protein; lactate dehydrogenase; C-reactive protein; activated partial thromboplastin time (aPTT), prothrombin time (PT), international normalized ratio (INR), D-dimer, and fibrinogen. Results of PLMS assays were generally reported directly into the EHR records of each participant, with a few exceptions which were reported as research data also contained in the VHA EHR. Assay results are documented and updated regularly on the CSP#2028 website: https://www.seattle.eric.research.va.gov/research/CSP-2028-EPIC3/home.asp.

### Data characteristics

Around 88.5% of participants have completed the study, 7.8% participants deceased and 3.7% withdrawn during follow-up (as of March 17th, 2025). Participants in the outpatient sub-cohort were younger than members of the inpatient and CLC sub-cohorts. The percentage of participants identifying as Black or African American in the inpatient, outpatient, and CLC sub-cohorts were approximately 39%, 18%, and 36%, respectively. Less than 10% of participants in the inpatient and CLC sub-cohorts identified as being Hispanic or Latino. A higher proportion of participants in the SARS-CoV-2 positive group were unvaccinated (< 1 dose) prior to their index date starting 1 month after a SARS-CoV-2 vaccine was first available to VHA users, compared with their negative counterparts (Table 2). Among the 131 CLC participants without SARS-CoV-2 infection at enrollment, 80 (61.1%) of them tested positive at least once during follow-up. Regarding the completion of specimen collections and questionnaires, the proportions of participants completing baseline questionnaires were 71.3%, 94.6%, and 84.9% in the inpatient, outpatient, and CLC sub-cohorts, respectively. The completion rate of vaccination questionnaire and symptoms questionnaire (flu-related) at baseline was similar among the three sub-cohorts. However, the completion rate of questionnaires in the outpatient sub-cohort became much higher than the other two sub-cohorts during follow-up. For biospecimens, the completion rate of blood collection was 67.8%, 15%, and 57.5% at baseline in the inpatient, outpatient, and CLC sub-cohorts, respectively. But the completion rate of blood in the outpatient sub-cohort exceeded the other sub-cohorts during follow-up. The trends remained similar for saliva and swabs. For either inpatient or outpatient sub-cohort, the completion rates of specimen collection and questionnaire were a little bit higher in the SARS-CoV-2 negative group compared with the positive group during follow-up (Supplementary Table 1). More information regarding the completion rates of biospecimen collection and questionnaire and their availability can be found on the official website with the page indicating research progress (https://www.seattle.eric.research.va.gov/research/CSP-2028-EPIC3/Study-progress.asp).

**Table 2:** Participants' demographic and clinical characteristics at baseline, stratified by sub-cohort and SARS-CoV-2 infection status.

	**Inpatient**		**Outpatient**		**CLC**	
**Characteristic**	**SARS-CoV-2 Negative**, ***N*** = **306**^a^	**SARS-CoV-2 Positive**, ***N*** = **430**^a^	**SARS-CoV-2 Negative**, ***N*** = **632**^a^	**SARS-CoV-2 Positive**, ***N*** = **1,280**^a^	**SARS-CoV-2 Negative**, ***N*** = **131**^a, b^	**SARS-CoV-2 Positive**, ***N*** = **55**^a, b^
**Study participation category** ^c^						
Enrolled (Completed Study)	241 (78.8%)	336 (78.1%)	581 (91.9%)	1,204 (94.1%)	102 (77.9%)	43 (78.2%)
Enrolled (Deceased)	59 (19.3%)	79 (18.4%)	28 (4.4%)	25 (2.0%)	23 (17.6%)	8 (14.6%)
Enrolled (Withdrawn)	6 (2.0%)	15 (3.5%)	23 (3.6%)	51 (4.0%)	6 (4.6%)	4 (7.3%)
**Age at enrollment**						
Median (IQR)	65.7 (57.1, 74.0)	67.3 (59.2, 74.4)	60.1 (46.3, 68.4)	54.4 (40.9, 66.2)	70.5 (64.2, 75.4)	71.4 (64.5, 75.9)
**Sex**						
Female	20 (6.5%)	39 (9.1%)	113 (18.0%)	233 (18.3%)	10 (7.6%)	3 (5.5%)
Male	286 (93.5%)	390 (90.9%)	513 (81.6%)	1,028 (80.7%)	118 (90.1%)	50 (90.9%)
Prefer no response/unknown	0 (0.0%)	1 (0.2%)	6 (0.9%)	19 (1.5%)	3 (2.3%)	2 (3.6%)
**Race**						
White alone	151 (49.4%)	226 (52.6%)	421 (66.6%)	828 (64.7%)	77 (58.8%)	25 (45.5%)
Black/African American alone	126 (41.2%)	162 (37.7%)	123 (19.5%)	225 (17.6%)	41 (31.3%)	26 (47.3%)
Other alone	7 (2.3%)	4 (0.9%)	18 (2.8%)	59 (4.6%)	1 (0.8%)	0 (0.0%)
Multiracial	12 (3.9%)	21 (4.9%)	63 (10.0%)	137 (10.7%)	11 (8.4%)	3 (5.5%)
Unknown	10 (3.3%)	17 (4.0%)	7 (1.1%)	31 (2.4%)	1 (0.8%)	1 (0.8%)
**Ethnicity being Hispanic or Latino**	28 (9.2%)	24 (5.6%)	73 (11.6%)	199 (15.5%)	10 (7.6%)	3 (5.5%)
**Vaccination status prior to index date**						
No vaccination	157 (51.3%)	308 (71.6%)	347 (54.9%)	801 (62.6%)	104 (79.4%)	40 (72.7%)
One dose	1 (0.3%)	7 (1.6%)	13 (2.1%)	34 (2.7%)	4 (3.1%)	2 (3.6%)
Complete dose	21 (6.9%)	37 (8.6%)	69 (10.9%)	142 (11.1%)	10 (7.6%)	0 (0.0%)
Complete plus booster	127 (41.5%)	78 (18.1%)	203 (32.1%)	303 (23.7%)	13 (9.9%)	13 (23.6%)
**Charlson comorbidity index** ^d^						
Median (IQR)	3.0 (1.0, 5.0)	3.0 (1.0, 5.0)	1.0 (0.0, 2.0)	0.0 (0.0, 2.0)	5.0 (3.0, 6.0)	4.00 (4.0, 6.0)

^a^n (%).

^b^All residents of participating CLCs were eligible to enroll regardless of their SARS-CoV-2 infection status at enrollment. The SARS-CoV-2 status of CLC participants at baseline were confirmed by SARS-CoV-2 RT-PCR, SARS-CoV-2 antibody, and SARS-CoV-2 antigen. Participants had any positive tests from the 21 days prior to index date to enrollment date were marked as positive at baseline, otherwise were marked as negative.

^c^Study participation category was updated most recently on March 17^th^, 2025. Enrolled (Completed Study) means participants were enrolled and have completed 2-year follow-up. Enrolled (Deceased) means participants were enrolled and deceased during follow-up. Enrolled (Withdrawn) means participants have officially withdrawn from the study or the site LSI (Local Site Investigator) has decided to withdraw the participants from the study for a specific reason (for example: safety reason). The study will not use the participants' electronic health records data past the date of withdrawal.

^d^Participants' comorbidities being considered in the study include: circulatory system problems (i.e., high blood pressure, stroke, coronary heart disease et al.), skeletal/muscular problems (i.e., osteoarthritis, rheumatoid arthritis et al.), mental health disorders (i.e., anxiety reaction/panic disorder, depression, eating disorder et al.), hearing/vision problems (i.e., cataracts, glaucoma et al.), infectious diseases (i.e., tuberculosis, hepatitis C et al.), kidney diseases, digestive system problems (i.e., acid reflux, ulcerative colitis et al.), cancer (i.e., breast, colon/rectal et al.), nervous system problems (i.e., migraine headaches et al.), other conditions (i.e., asthma, chronic lung disease, diabetes et al.).

## Strengths and limitations of EPIC^3^ database

### Strengths

The EPIC^3^ database is a valuable resource for investigating the natural history of SARS-CoV-2 infection and subsequent health, as well as risk factors associated with infection and virus evolvement since participants were recruited from diverse settings (inpatient, outpatient, and community living centers) across the U.S and with rich resources for data collection, including questionnaires, biospecimens, and electronic health records (EHRs). Validity and reliability is another great asset of the EPIC^3^ database. Participants' SARS-CoV-2 statuses at baseline are strictly ascertained by laboratory test results and questionnaire data/biospecimens are collected by externally validated instruments with systematic quality control activities. In addition, information collected from questionnaires and EHRs can cross-validate with each other.

### Limitations

There are several limitations we would like to note for utilizing EPIC^3^ resources. First, there are potential issues with loss-to-follow-up (LTFU) or data missingness at certain time periods. However, LTFU is likely not a serious threat to internal validity since the rate of withdrawal from the study was minimal (Table 2). Issues with data missingness at various time periods may be dealt with by design. For example, missing questionnaire data may be supplemented with comparable EHR records. In addition, the indefinite extraction of EHR data allows participants to be longitudinally followed-up well past the initial 24-month study period. Similarly, specimens banked at the biorepository may be assayed for markers not initially specified at the time of enrollment, rate limited by collected sample quantity, allowing for flexibility to respond to emerging research questions. We would also like to note that Veterans may receive care at other institutions and if they were over 65 years old, some of their data may be available through Centers for Medicare and Medicaid Services instead of EHRs at VHA.

Second, SARS-CoV-2 positive participants were not required to be symptomatic with COVID-like illness for recruitment purposes, resulting in heterogeneity in their health conditions and the associated indication for testing. But given the rich diverse data repositories in this study, investigators are welcome to manipulate the dataset and generate indicators (i.e., new definitions of ascertaining SARS-CoV-2 status) according to their research goals.

Third, the allotted recruitment timeframe differed between SARS-CoV-2 positive and negative participants. Positives were required to be recruited within 72 h of identification from CDW records, while only an approximately similar timeframe was recommended for negatives. The amount of time between initial recruitment and final enrollment varied among participants as well.

Lastly, issues of potential selection bias and generalizability should be considered. In EPIC^3^, there is a much higher proportion of biological men than women, making it challenging to study the effect of sex. In addition, the patterns of receiving services at VHA medical centers differ in various phases of the pandemic/recruitment period, due to transitions in hospital access restrictions, quarantine mandates, conversion of in-person appointments to telehealth visits, and availability of COVID-19 testing (34). This may indicate systematic differences between enrolled participants and the corresponding population to whom we desire to generalize results. However, given the relatively long recruitment period and that many participants were recruited during later phases of the pandemic, potential selection bias issues should be minimal.

## Data Availability

The raw data supporting the conclusions of this article will be made available by the authors in accordance with VA policy.
