# Supplementary material for: Epidemiology, Immunology and Clinical Characteristics of COVID-19 (EPIC3)—Database of a prospective longitudinal observational study within the Veterans Health Administration
Source: Front Public Health. 2025 Jun 2;13:1535315. doi: 10.3389/fpubh.2025.1535315 (PMC12178061; doi:10.3389/fpubh.2025.1535315)
Supplement: Supplementary file 1 [file Data_Sheet_1.pdf]

Supplementary Table 1. Summarization of the participants completing biospecimen collections and questionnaire at each visit, stratifying by sub-cohort and SARS-CoV-2 statuses

		Day 0[a]	Day 3[a]	Day 7[a]	Day 14[a]	Day 21[a]	Day 28[a]	Month 3[a]	Month 6[a]	Month 9[a]	Month 12[a]	Month 15[a]	Month 18[a]	Month 21[a]	Month 24[a]
**Inpatient sub-cohort**															
**SARS-CoV-2 Positive (N = 430)**	Blood[b]	288 (67.0%)	88 (20.5%)	76 (17.7%)	62 (14.4%)	71 (16.5%)	118 (27.4%)	113 (26.3%)	136 (31.6%)	-	140 (32.6%)	-	123 (28.6%)	-	127 (29.5%)
	Saliva[c]	38 (8.8%)	34 (7.9%)	43 (10.0%)	52 (12.1%)	57 (13.3%)	78 (18.1%)	63 (14.7%)	71 (16.5%)	-	81 (18.8%)	-	53 (12.3%)	-	66 (15.3%)
	Swab[d, e]	95 (22.1%)	33 (7.7%)	23 (5.3%)	18 (4.2%)	24 (5.6%)	37 (8.6%)	28 (6.5%)*	28 (6.5%)*	-	44 (10.2%)*	-	40 (9.3%)*	-	21 (4.9%)*
	Baseline Survey	310 (72.1%)	-	-	-	-	-	-	-	-	-	-	-	-	-
	Flu-related Symptoms Survey	347 (80.7%)	155 (36.0%)	148 (34.4%)	148 (34.4%)	138 (32.1%)	189 (44.0%)	175 (40.7%)	195 (45.3%)	-	203 (47.2%)	-	186 (43.3%)	-	191 (44.4%)
	Long Term Symptoms Survey	-	110 (25.6%)	121 (28.1%)	130 (30.2%)	116 (27.0%)	166 (38.6%)	169 (39.3%)	192 (44.7%)	-	200 (46.5%)	-	184 (42.8%)	-	190 (44.2%)
	Vaccine Survey	291 (67.7%)	131 (30.5%)	127 (29.5%)	133 (30.9%)	120 (27.9%)	171 (39.8%)	169 (39.3%)	194 (45.1%)	-	201 (46.7%)	-	188 (43.7%)	-	191 (44.4%)
**SARS-CoV-2 Negative (N = 306)**	Blood[b]	211 (69.0%)	73 (23.9%)	68 (22.2%)	61 (19.9%)	62 (20.3%)	106 (34.6%)	120 (39.2%)	121 (39.5%)	-	125 (40.8%)	-	106 (34.6%)	-	117 (38.2%)
	Saliva[c]	37 (12.1%)	32 (10.5%)	40 (13.1%)	43 (14.1%)	47 (15.4%)	68 (22.2%)	65 (21.2%)	57 (18.6%)	-	63 (20.6%)	-	62 (20.3%)	-	48 (15.7%)
	Swab[d, e]	89 (29.1%)	37 (12.1%)	44 (14.4%)	36 (11.8%)	39 (12.7%)	50 (16.3%)	30 (9.8%)*	41 (13.4%)*	-	36 (11.8%)*	-	30 (9.8%)*	-	25 (8.2%)*
	Baseline Survey	215 (70.3%)	-	-	-	-	-	-	-	-	-	-	-	-	
	Flu-related Symptoms Survey	246 (80.4%)	110 (35.9%)	112 (36.6%)	109 (35.6%)	119 (38.9%)	148 (48.4%)	152 (49.7%)	154 (50.3%)	-	162 (52.9%)	-	151 (49.3%)	-	152 (49.7%)
	Long Term Symptoms Survey	-	83 (27.1%)	96 (31.4%)	104 (34.0%)	109 (35.6%)	140 (45.8%)	141 (46.1%)	149 (48.7%)	-	162 (52.9%)	-	149 (48.7%)	-	152 (49.7%)
	Vaccine Survey	219 (71.6%)	92 (30.1%)	102 (33.3%)	103 (33.7%)	111 (36.3%)	143 (46.7%)	143 (46.7%)	154 (50.3%)	-	161 (52.6%)	-	151 (49.3%)	-	152 (49.7%)
**Outpatient sub-cohort**														-	
**SARS-CoV-2 Positive (N = 1280)**	Blood[b]	100 (7.8%)	247 (19.3%)	329 (25.7%)	397 (31.0%)	398 (31.1%)	519 (40.5%)	412 (32.2%)	443 (34.6%)	-	405 (31.6%)		430 (33.6%)	-	424 (33.1%)
	Saliva[c]	102 (8.0%)	301 (23.5%)	374 (29.2%)	465 (36.3%)	441 (34.5%)	562 (43.9%)	422 (33.0%)	461 (36.0%)	-	414 (32.3%)	-	428 (33.4%)	-	442 (34.5%)
	Swab[d, e]	26 (2.0%)	24 (1.9%)	61 (4.8%)	95 (7.4%)	97 (7.6%)	136 (10.6%)	21 (1.6%)*	7 (0.5%)*	-	40 (3.1%)*	-	14 (1.1%)*	-	11 (0.9%)*
	Baseline Survey	1208 (94.4%)	-	-	-	-	-	-	-	-	-	-	-	-	-
	Flu-related Symptoms Survey	967 (75.5%)	857 (67.0%)	928 (72.5%)	916 (71.6%)	890 (69.5%)	1004 (78.4%)	897 (70.1%)	909 (71.0%)	-	845 (66.0%)	-	854 (66.7%)	-	866 (67.7%)
	Long Term Symptoms Survey	-	766 (59.8%)	892 (69.7%)	890 (69.5%)	867 (67.7%)	988 (77.2%)	895 (69.9%)	905 (70.7%)	-	840 (65.6%)	-	843 (65.9%)	-	858 (67.0%)

	Vaccine Survey	947 (74.0%)	834 (65.2%)	912 (71.3%)	895 (69.9%)	875 (68.4%)	991 (77.4%)	897 (70.1%)	908 (70.9%)	-	843 (65.9%)	-	853 (66.6%)	-	863 (67.4%)
**SARS-CoV-2 Negative (N = 632)**	Blood[b]	186 (29.4%)	185 (29.3%)	242 (38.3%)	272 (43.0%)	251 (39.7%)	327 (51.7%)	270 (42.7%)	248 (39.2%)	-	242 (38.3%)	-	228 (36.1%)	-	212 (33.5%)
	Saliva[c]	148 (23.4%)	183 (29.0%)	233 (36.9%)	256 (40.5%)	240 (38.0%)	308 (48.7%)	251 (39.7%)	239 (37.8%)	-	219 (34.7%)	-	207 (32.8%)	-	204 (32.3%)
	Swab[d, e]	141 (22.3%)	94 (14.9%)	116 (18.4%)	124 (19.6%)	113 (17.9%)	146 (23.1%)	11 (1.7%)*	6 (0.9%)*	-	17 (2.7%)*	-	6 (0.9%)*	-	3 (0.5%)*
	Baseline Survey	600 (94.9%)								-		-		-	
	Flu-related Symptoms Survey	504 (79.7%)	465 (73.6%)	494 (78.2%)	503 (79.6%)	476 (75.3%)	527 (83.4%)	492 (77.8%)	445 (70.4%)	-	428 (67.7%)	-	427 (67.6%)	-	420 (66.5%)
	Long Term Symptoms Survey	-	426 (67.4%)	479 (75.8%)	497 (78.6%)	471 (74.5%)	526 (83.2%)	490 (77.5%)	443 (70.1%)	-	425 (67.2%)	-	424 (67.1%)	-	419 (66.3%)
	Vaccine Survey	504 (79.7%)	460 (72.8%)	491 (77.7%)	500 (79.1%)	471 (74.5%)	527 (83.4%)	492 (77.8%)	445 (70.4%)	-	425 (67.2%)	-	426 (67.4%)	-	420 (66.5%)
**CLC sub-cohort**															
**SARS-CoV-2 Positive (N = 55)**	Blood[b]	34 (61.8%)	-	-	-	-	-	14 (25.5%)	21 (38.2%)	8 (14.5%)	26 (47.3%)	9 (16.4%)	23 (41.8%)	8 (14.5%)	18 (32.7%)
	Saliva[c]	2 (3.6%)*	-	-	-	-	-	1 (1.8%)*	5 (9.1%)*	(0.0%)*	13 (23.6%)*	1 (1.8%)*	9 (16.4%)*	(0.0%)*	10 (18.2%)*
	Swab[d, e]	3 (5.5%)*	-	-	-	-	-	2 (3.6%)*	5 (9.1%)*	3 (5.5%)*	3 (5.5%)*	4 (7.3%)*	3 (5.5%)*	3 (5.5%)*	1 (1.8%)*
	Baseline Survey	44 (80.0%)	-	-	-	-	-	-	-	-	-	-	-	-	-
	Flu-related Symptoms Survey	41 (74.5%)	-	-	-	-	-	31 (56.4%)	28 (50.9%)	19 (34.5%)	32 (58.2%)	15 (27.3%)	30 (54.5%)	13 (23.6%)	26 (47.3%)
	Long Term Symptoms Survey	-	-	-	-	-	-	30 (54.5%)	28 (50.9%)	19 (34.5%)	31 (56.4%)	15 (27.3%)	30 (54.5%)	13 (23.6%)	25 (45.5%)
	Vaccine Survey	41 (74.5%)	-	-	-	-	-	31 (56.4%)	30 (54.5%)	19 (34.5%)	33 (60.0%)	15 (27.3%)	30 (54.5%)	13 (23.6%)	25 (45.5%)
**SARS-CoV-2 Negative (N = 131)**	Blood[b]	73 (55.7%)	-	-	-	-	-	38 (29.0%)	38 (29.0%)	32 (24.4%)	40 (30.5%)	19 (14.5%)	37 (28.2%)	18 (13.7%)	34 (26.0%)
	Saliva[c]	2 (1.5%)*	-	-	-	-	-	1 (0.8%)*	8 (6.1%)*	9 (6.9%)*	18 (13.7%)*	(0.0%)*	16 (12.2%)	2 (1.5%)	18 (13.7%)
	Swab[d, e]	9 (6.9%)*	-	-	-	-	-	11 (8.4%)*	9 (6.9%)*	10 (7.6%)*	10 (7.6%)*	11 (8.4%)*	8 (6.1%)*	3 (2.3%)*	(0.0%)*
	Baseline Survey	114 (87.0%)	-	-	-	-	-	-	-	-	-	-	-	-	-
	Flu-related Symptoms Survey	104 (79.4%)	-	-	-	-	-	75 (57.3%)	57 (43.5%)	47 (35.9%)	63 (48.1%)	29 (22.1%)	64 (48.9%)	24 (18.3%)	55 (42.0%)
	Long Term Symptoms Survey	-	-	-	-	-	-	75 (57.3%)	57 (43.5%)	46 (35.1%)	63 (48.1%)	29 (22.1%)	63 (48.1%)	24 (18.3%)	55 (42.0%)
	Vaccine Survey	106 (80.9%)	-	-	-	-	-	76 (58.0%)	58 (44.3%)	47 (35.9%)	63 (48.1%)	29 (22.1%)	64 (48.9%)	24 (18.3%)	56 (42.7%)

a. n(%)

b. Blood collections include one or more biospecimens of the following types: Peripheral blood mononuclear cells (PBMC), Ethylenediaminetetraacetic acid (EDTA) Plasma, Sodium Citrate Plasma, Sodium Citrate Buffy Coat, PAXgene Blood RNA Tube, Serum (venous), Serum (capillary)

c. Saliva collections include one or more biospecimens of the following types: Saliva (with preservative), Saliva (without preservative). For CLC sub-cohort, saliva was not required for any visit and the saliva were provided as clinical residual samples when available, which is noted as * in the table

d. Swab collections include one or more biospecimens of the following types: Nasal swab, Nasopharyngeal swab, Oropharyngeal swab

e. Swabs were only required for up to 6 visits (Day 0 – Day 28) for Inpatient and Outpatient sub-cohorts, and swabs were not required for any visit of the CLC sub-cohort. However, sometimes additional swabs were provided as clinical residual samples when available, which is noted as * in the table

\- Means the specimen or questionnaire is not requested for that visit
